# Optimizing robotic thyroid surgery: lessons learned from an retrospective analysis of 104 cases

**DOI:** 10.3389/fendo.2024.1337322

**Published:** 2024-01-30

**Authors:** Bo Wang, Jia-Fan Yu, Wei Ao, Jun Wang, Xin-Yi Guo, Meng-Yao Li, Wen-Yu Huang, Chi-Peng Zhou, Shou-Yi Yan, Li-Yong Zhang, Si-Si Wang, Shao-Jun Cai, Si-Ying Lin, Wen-Xin Zhao

**Affiliations:** ^1^ Department of Thyroid Surgery, Fujian Medical University Union Hospital, Fujian, Fuzhou, China; ^2^ Clinical Research Center for Precision Management of Thyroid Cancer of Fujian Province, Fuzhou, China

**Keywords:** robotic thyroidectomy, da Vinci surgical system, learning curve, operative efficiency, postoperative outcomes, thyroid surgery

## Abstract

**Background:**

Robotic assistance in thyroidectomy is a developing field that promises enhanced surgical precision and improved patient outcomes. This study investigates the impact of the da Vinci Surgical System on operative efficiency, learning curve, and postoperative outcomes in thyroid surgery.

**Methods:**

We conducted a retrospective cohort study of 104 patients who underwent robotic thyroidectomy between March 2018 and January 2022. We evaluated the learning curve using the Cumulative Sum (CUSUM) analysis and analyzed operative times, complication rates, and postoperative recovery metrics.

**Results:**

The cohort had a mean age of 36 years, predominantly female (68.3%). The average body mass index (BMI) was within the normal range. A significant reduction in operative times was observed as the series progressed, with no permanent hypoparathyroidism or recurrent laryngeal nerve injuries reported. The learning curve plateaued after the 37th case. Postoperative recovery was consistent, with no significant difference in hospital stay duration. Complications were minimal, with a noted decrease in transient vocal cord palsy as experience with the robotic system increased.

**Conclusion:**

Robotic thyroidectomy using the da Vinci system has demonstrated a significant improvement in operative efficiency without compromising safety. The learning curve is steep but manageable, and once overcome, it leads to improved surgical outcomes and high patient satisfaction. Further research with larger datasets and longer follow-up is necessary to establish the long-term benefits of robotic thyroidectomy.

## Introduction

The advent of robotic assistance in the realm of surgery has catalyzed a paradigm shift in the way operative procedures are performed, particularly in thyroid surgery. The precision and flexibility offered by robotic systems such as the da Vinci Surgical System have the potential to overcome limitations associated with traditional and endoscopic thyroidectomy techniques, offering a new horizon for surgical innovation (1). This study aims to evaluate the efficacy, learning curve, and safety profile of robotic thyroidectomy in a comprehensive cohort of 104 cases performed over a span of nearly four years.

Thyroidectomy, a common surgical procedure for the treatment of thyroid nodules and cancer, has traditionally been associated with specific challenges and complications, such as hypoparathyroidism and recurrent laryngeal nerve (RLN) injury. The intricate nature of thyroid anatomy requires meticulous dissection and preservation of vital structures, demanding high surgical skill and experience. Robotic thyroidectomy promises to mitigate such risks through enhanced dexterity, three-dimensional visualization, and ergonomic advantage, facilitating more precise and less invasive surgeries (2).

Despite its benefits, the adoption of robotic thyroidectomy has been gradual, mainly due to the steep learning curve associated with mastering the robotic system (3). This learning curve can impact operative times and potentially influence complication rates. Hence, it is imperative to analyze the progression of surgical efficiency as the operative team becomes more adept with the technology (4). Moreover, the impact of robotic assistance on postoperative outcomes, including patient recovery time and complication rates, needs thorough investigation to establish the advantages over traditional methods.

In this retrospective cohort study, we meticulously reviewed 104 robotic thyroidectomy cases, focusing on adults with malignant thyroid nodules, to delineate the learning curve and to assess the operative and postoperative outcomes. We hypothesized that despite the initial investment in learning and training, the da Vinci Surgical System would facilitate a decrease in operative times, with a concomitant maintenance of low complication rates, thus enhancing overall patient care in thyroid surgery. Our study contributes to the existing literature by providing a detailed analysis of the transition phase from traditional to robotic thyroidectomy, emphasizing the importance of strategic training and systematic application in achieving surgical proficiency with robotic systems (5).

## Methods

### Study design and participant criteria

This retrospective cohort study meticulously assessed 104 cases of robotic thyroidectomy performed from March 2018 to January 2022. Strict inclusion criteria selected for adult patients aged 18-60 with malignant thyroid nodules smaller than 4 cm, with or without lymph node or distant metastasis, ensuring a representative patient population. Cases involving tumor invasion into surrounding tissues, lateral lympha node metastasis, a history of neck surgery, or contraindications to general anesthesia were excluded to maintain a homogeneous sample and integrity of the outcome measures. all surgeries within this study were consistently performed by the same surgeon, Professor Wen-Xin Zhao, ensuring uniformity in surgical technique and approach.

### Preoperative assessment

High-resolution ultrasonography and fine-needle aspiration biopsies, with or without genetic analysis for BRAF V600E mutations and RET gene rearrangements, constituted the preoperative evaluation. Uniform laryngoscopic examination was performed across the cohort to confirm vocal cord functionality, which was critical for tailoring the surgical approach and minimizing operative risk.

### Robotic surgical methodology

The surgeries employed the da Vinci Surgical System, utilizing both Si and Xi models in sequence. The initial 24 cases were performed with the Si model, after which the models were alternated weekly. The Bilateral Axillo-Breast Approach (BABA) was uniformly adopted for its direct access and cosmetic benefits, contributing to the consistency in surgical outcomes as reported (6, 7).

### Surgical technique

Under general anesthesia, patients were positioned to enhance access to the thyroid region. The BABA technique provided a standardized surgical approach, including flap creation (8), trocar insertion, and robotic arm docking, aligning with the uniform procedural methodology that resulted in no cases of permanent hypoparathyroidism or recurrent laryngeal nerve injuries. Parathyroid glands not viable for preservation *in situ* underwent autotransplantation, reflecting the stable postoperative PTH levels seen across the cohort (9, 10).

### Postoperative management

A regimented postoperative care protocol was implemented, focusing on pain management, early mobilization, and hypocalcemia monitoring. Oral calcium supplementation was provided until serum PTH levels normalized, mirroring the consistent and favorable postoperative course documented in the results (11).

### Statistical analysis

Statistical evaluations were performed utilizing SPSS version 29.0, which facilitated the analysis of operative times, complication types, numbers of lymph nodes dissected, duration of hospital stays, and essential biochemical indicators including parathyroid hormone (PTH) and calcium levels. The learning curve associated with the surgical procedures was assessed using Cumulative Sum (CUSUM) analysis. Comprehensive statistical comparisons were executed within SPSS, with a p-value threshold of <0.05 established for the determination of statistical significance.

## Results

### Demographic and preoperative data

The current study evaluated 104 patients who underwent robotic thyroidectomy, with a female majority (71 cases, 68.3%) and the remainder male (33 cases, 31.7%). The average age was established at 36 years with a standard deviation of 8.12 years. The body mass index (BMI) for the collective was a mean of 22.50 with a standard deviation of 3.34, placing the majority in a normal weight category. The pathology was confined to papillary thyroid carcinoma with 97 instances of the classic variant, 8 of the follicular subtype, and 2 cases presenting with both variants. The mean tumor size was relatively small at 0.94 cm in diameter with a standard deviation of 0.64 cm. Each surgical procedure was completed as planned, without the need for conversion to open or endoscopic-assisted methods, and there were no reports of permanent hypoparathyroidism or recurrent laryngeal nerve injuries. These demographic and clinical features are extensively outlined in **Table 1**.

**Table 1:** Demographics and Surgical Outcomes.

Characteristics	Number or Mean ± SD
**N**	104
**Gender (Male/Female)**	33/71
**Age (mean ± SD)**	35.97 ± 8.12
**BMI (mean ± SD)**	22.50 ± 3.34
**DaVinci Robot Model (Si/Xi)**	51/53
**Pathology Variation Classic/Follicular**	97/8(2*)
**Hemi/Total Thyroidectomy**	85/19
**Tumor Diameter (mean ± SD)**	0.94 ± 0.64
**Operation Durations (mean ± SD)**	163.59 ± 39.38
**Drainage(ml)**	175.73 ± 64.04
**Postoperative Hospitalization days (mean ± SD)**	2.94 ± 0.87

*Two patients with multifocal cancer, both classic and follicular subtypes.

### Operative efficiency and learning curve outcomes

**Figure 1:**
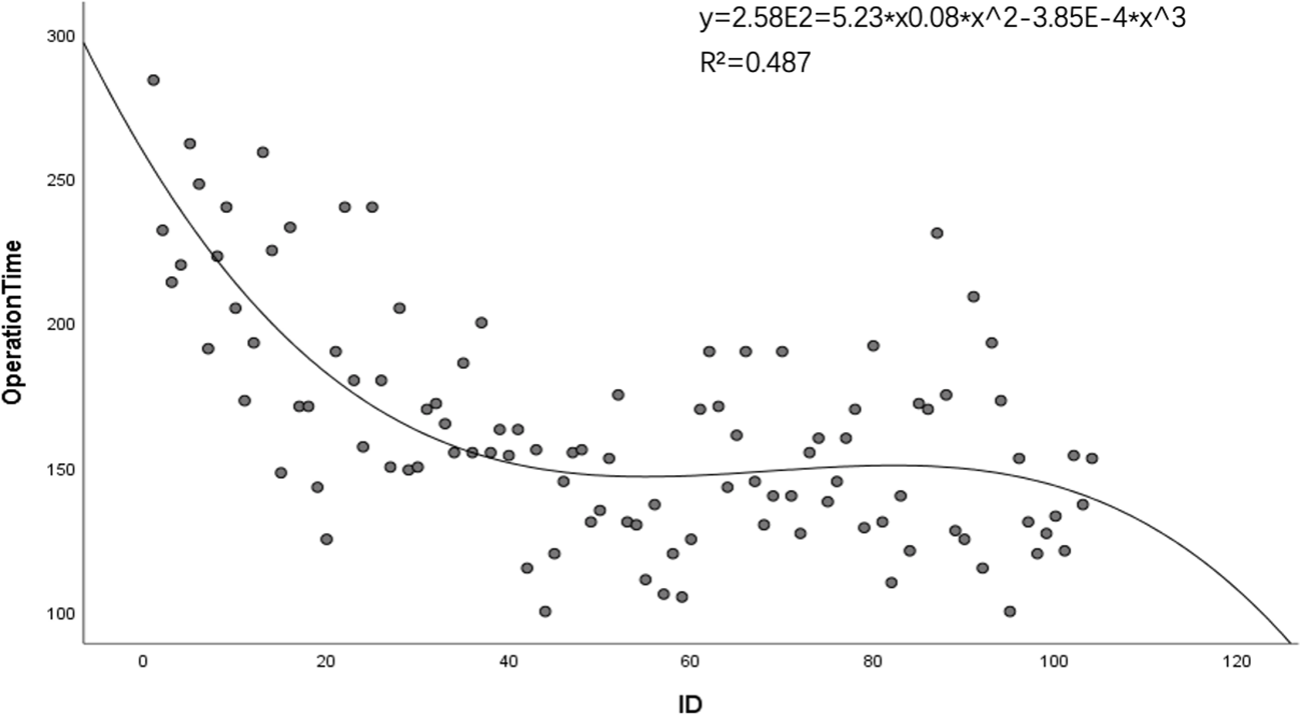
Trend analysis of operative durations for robotic thyroidectomy performed using the bilateral axillo-breast approach.

**Figure 2:**
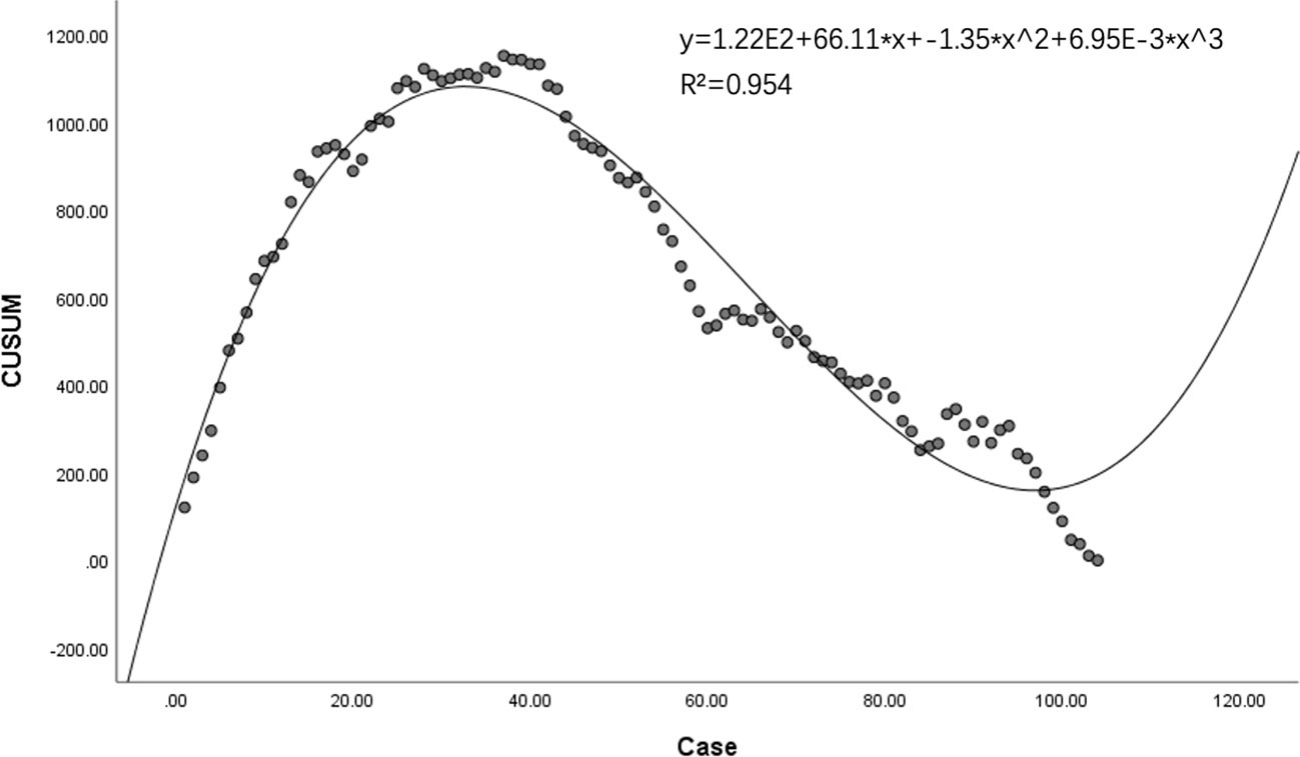
Proficiency progression in robotic thyroidectomy: a CUSUM analysis depicting learning curve dynamics.

A significant decline in mean operative durations was noted, decreasing from 194 minutes (SD = 39 minutes) for the initial group of 37 cases to 146 minutes (SD = 27 minutes) for the latter 67 cases (p = 0.020). The mean drainage volume post-operation remained consistent at 175.73 ml (SD = 64.04 ml). Cumulative sum (CUSUM) analysis suggested that a significant learning threshold was surpassed subsequent to the 37th case. This is visually represented in **Figures 1**, **2**. Despite reaching technological proficiency and harvesting an additional two lymph nodes on average, the difference did not reach statistical significance (P=0.105). For detailed data, see **Table 2**.

**Table 2:** Comparative Analysis of Surgical Proficiency.

Parameters	Learning Improvement Period	Skill Mastery Period	P Value
**N**	37	67	NA
**Gender (Male/Female)**	8/29	25/42	0.100
**Age (mean ± SD)**	35.51 ± 8.80	36.22 ± 7.79	0.654
**BMI (mean ± SD)**	22.01 ± 3.25	22.70 ± 3.37	0.338
**DaVinci Robot Model (Si/Xi)**	27/10	24/43	0.001
**Hemi/Total Thyroidectomy**	29/8	44/23	0.175
**Tumor Diameter (mean ± SD)**	1.09 ± 0.82	0.88 ± 0.56	0.020
**Operation durations (mean ± SD)**	194.70 ± 39.43	146.40 ± 27.02	0.020
**Postoperative Hospitalization days (mean ± SD)**	3.08 ± 0.95	2.87 ± 0.82	0.228
**Postoperative Drainage Volume (mean ± SD)**	161 ± 70.62	183 ± 59.13	0.831
**Parathyroid Glands In Situ(case)**	9/37	10/67	0.291
**Parathyroid Glands Transplanted(case)**	28/37	57/67	0.235
**Parathyroid Glands Inadvertently Removed(number**, **Percentage)**	4/37(10.8%)	4/67(5.97%)	0.375
**Total Number of Lymph Nodes Harvested**	9.84 ± 5.93	11.93 ± 6.39	0.105
**Lymph Nodes with Metastasis**	1.62 ± 2.64	2.22 ± 3.43	0.357
**PTH Pre OP (pmol/L)**	4.79 ± 2.10	4.22 ± 1.71	0.154
**PTH day 1 Post OP (pmol/L)**	2.40 ± 1.28	2.04 ± 1.19	0.173
**PTH day 7 Post OP (pmol/L)**	3.20 ± 1.84	3.06 ± 1.97	0.799
**PTH day 30 Post OP (pmol/L)**	3.94 ± 2.19	4.20 ± 1.77	0.664
**Ca^2+^ Pre OP (mmol/L)**	2.31 ± 0.10	2.32 ± 0.09	0.821
**Ca^2+^ day 1 Post OP (mmol/L)**	2.21 ± 0.12	2.25 ± 0.10	0.091
**Ca^2+^ day 7 Post OP (mmol/L)**	2.37 ± 0.10	2.32 ± 0.13	0.167
**Ca^2+^ day 30 Post OP (mmol/L)**	2.39 ± 0.13	2.30 ± 0.11	0.021
**Pathology Variation Classic/Follicular**	36/1	68/8(2*)	0.124

In our cohort, the reference ranges are PTH: 1.3 to 9.3 pmol/L and calcium: 2.1 to 2.7 mmol/L. NA, Not Applicable.

### Postoperative recovery and hospitalization metrics

**Figure 3:**
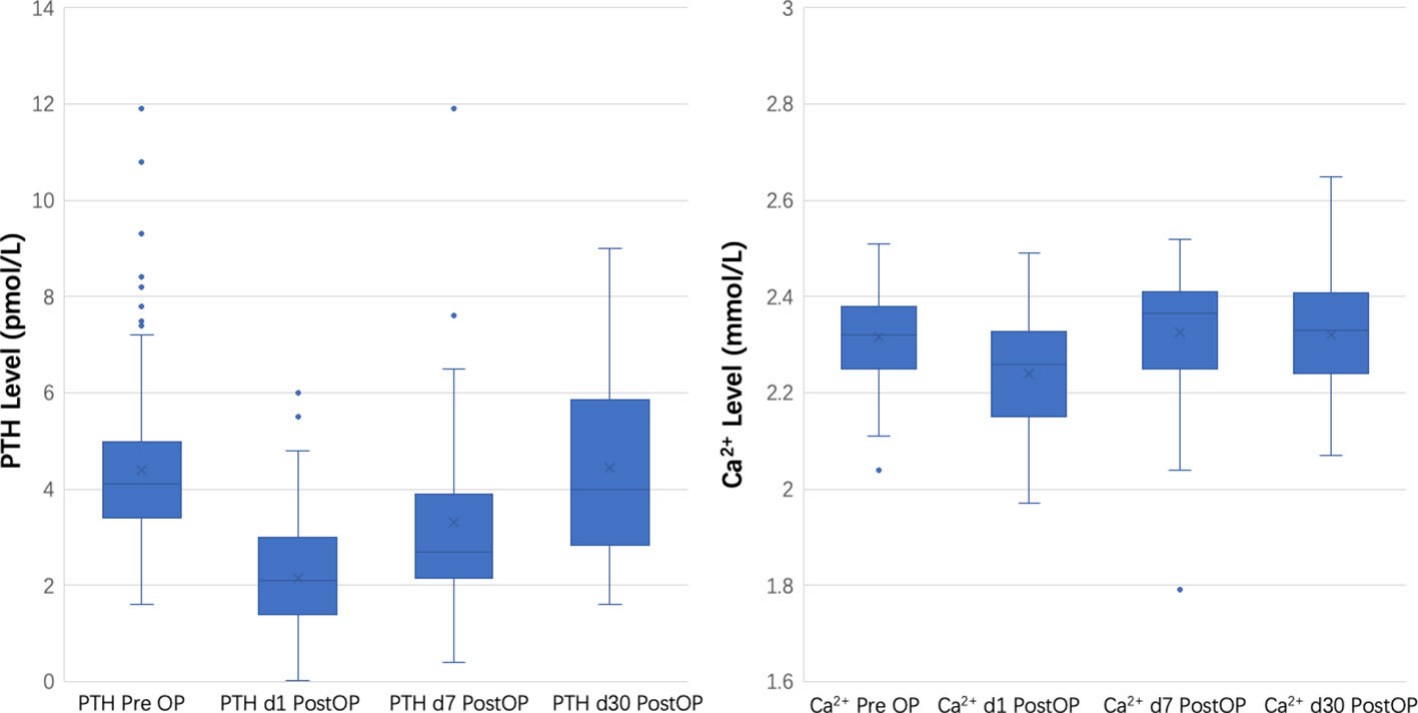
Comparative analysis of parathyroid hormone and serum calcium concentrations pre- and post-robotic thyroid surgery.

The duration of hospital stay post-thyroidectomy was consistent, with an overall mean of 2.94 days. The comparison between the initial learning phase and the subsequent mastery phase did not reveal significant differences (3.08 days, SD = 0.95 vs. 2.87 days, SD = 0.82; p = 0.228). The incidence of transient hypoparathyroidism did not significantly differ between the two periods; however, a reduction in inadvertent parathyroid gland excisions was noted (10.8% reduced to 5.97%, P=0.375), albeit without statistical significance. No instances of permanent hypoparathyroidism occurred. The consistency in the surgical approach is further corroborated by the lack of significant differences in postoperative parathyroid hormone and serum calcium levels between the groups, as depicted in **Figure 3**.

### Complications and surgical management

Postoperative complications were minimal and predominantly transient. The incidence of transient vocal cord palsy decreased from 5.4% (2/37) in the initial cases to 1.5% (1/67) in the latter cases. There were no occurrences of permanent recurrent laryngeal nerve injury or permanent hypoparathyroidism. Single instances of postoperative hemorrhage and tracheal injury were recorded, both during the early phase of the learning curve. Comprehensive complication rates are presented in **Table 3**.

**Table 3:** Surgical Complication Rates by Training Phase.

Parameters	Learning Improvement Period	Skill Mastery Period
**N**	37	67
**Transient Hypoparathyroidism**	7/36 (18.9%)	15/67 (22.4%)
**Permanent Hypoparathyroidism**	0	0
**Transient Recurrent Laryngeal Nerve Palsy**	2/37 (5.4%)	1/67 (1.5%)
**Permanent Recurrent Laryngeal Nerve Palsy**	0	0
**Bleeding**	1/37 (2.7%)	0
**Tracheal Injury**	1/37 (2.7%)	0

## Discussion

The integration of robotic technology into thyroid surgery has been a game-changer, reshaping both methods and results in the field. The analysis of our comprehensive series of 104 robotic thyroidectomy procedures echoes the broader scientific consensus, showcasing the pivotal role of the da Vinci Surgical System in enhancing the precision and overall effectiveness of these complex surgeries (12, 13). The data gleaned from our cohort clearly delineates a proficiency benchmark, which is in harmony with the well-established learning curve intrinsic to robotic surgical systems (14). This finding highlights the requisite investment in both time and specialized training that is essential for achieving a level of proficiency that translates to optimal surgical outcomes.

Our study documents a tangible decrease in operative times, which inversely correlates with the surgeons’ escalating experience—this trend is consistent with the evolution of surgical advancements highlighted in contemporary research (15). It’s also worth noting that our study extends beyond mere operative efficiency; it underscores the heightened safety profile of robotic thyroidectomy. The absence of any cases of permanent hypoparathyroidism or recurrent laryngeal nerve injury within our study population not only corroborates the high safety standards of the procedure but also serves as a testament to the reliability of robotic assistance when wielded by well-trained surgical hands (16). This evidence reinforces the notion that robotic thyroidectomy, when performed by skilled surgeons, is not only innovative but also a reliably safe intervention, setting a new benchmark for patient teatment in thyroid surgery (17).

Our initial experience with the cohort did present a small number of postoperative complications, including a single incident each of postoperative bleeding and tracheal injury. It is noteworthy that the occurrence of postoperative hemorrhage, at a rate of 0.9% (1/104), is notably lower than typically documented in traditional and endoscopic thyroid surgeries (13). However, the instance of tracheal injury, while rare in the realm of robotic surgeries, did manifest in our series. This particular case emerged one week postoperatively, with the patient presenting with cervical and prethoracic emphysema, but notably without dyspnea.

In an adept response, we immediately instituted a treatment protocol involving the placement of a drainage tube at the site of the emphysema, followed by central negative pressure suction and comprehensive anti-inflammatory therapy, leading to the patient’s full recovery. Further scrutiny revealed that the tracheal injury was likely linked to tumor invasion at the site of Berry’s ligament, an insight that has since informed our surgical approach and risk assessment. This case underscores the importance of vigilance and prompt intervention in managing such rare but significant complications and reflects our commitment to continuously refining our surgical practices in light of emerging evidence (18).

Navigating the shift from endoscopic to robotic thyroid surgery is a critical early challenge for practitioners adopting this advanced technology. Reflecting on over a hundred cases, we’ve distilled the transition into four essential adaptations for newcomers:

1. **Instrumental Dynamics:** Understanding the mechanical differences between instruments is crucial. Robotic tools feature a triple-joint configuration, unlike the double-joint nature of endoscopic instruments. This additional joint necessitates careful consideration of the instrument’s wrist posture to prevent friction against neck muscles beyond the surgical field (19).
2. **Assistant Coordination:** With the surgeon physically separated from the assistant, clear communication becomes vital. Assistants must be continually cognizant of the internal and external traction forces at play to minimize stress on delicate tissues, particularly before the surgeon has established a visual and mechanical feedback loop (20).
3. **Robotic Rhythmicity:** Surgeons must master the coordination of the camera, instrument spatial orientation, and arm posture, developing a robotic-specific rhythm for movements. This includes positioning, focusing, and resetting the surgical instruments and surgeon’s hand position for each stage of the procedure. Training to embed these actions into muscle memory is essential for fluid operation (21).
4. **Instrument Rotation Management:** Given the robotic instruments’ enhanced flexibility, allowing for 540 degrees of rotation compared to the human wrist’s 360 degrees, it is important to frequently reset tools, particularly the ultrasonic scalpel, to their initial position (marked at 270 degrees). This practice ensures harmony between the surgeon’s hand movements and the robotic instruments (22).

The main constraint of this study is its small sample size and the short duration of follow-up, which may limit the ability to firmly establish statistical significance for some results, especially in relation to the handling of parathyroid glands during thyroid surgery. Additionally, a comparative analysis was not conducted between different thyroidectomy approaches, such as transoral and axillary approaches, which could provide further insights. While a reduction in surgery duration was noted, it wasn’t accompanied by statistically significant reductions in complication rates. This suggests that the improvements may be more attributable to growing familiarity with the robotic equipment than to the surgical method itself (20). With a larger pool of data in the future, further decreases in surgery time might be observed, possibly reflecting refinements in surgical techniques like precise membrane dissection and better conservation of the parathyroid glands’ and RLN functionality (21). Cost is a notable concern in robotic thyroid surgery, yet recent advancements offer optimism. Newer robotic systems have reduced costs by up to 30%, indicating that the financial barrier to robotic surgery adoption may soon become less significant.

## Conclusions

The application of robotic technology in thyroid surgery, specifically using the da Vinci Surgical System, marks a significant shift towards more precise and patient-focused procedures. Our study’s findings, drawn from an analysis of 104 cases, reveal that the initial complexity of learning robotic thyroidectomy is a worthwhile investment leading to superior surgical precision and consistent patient outcomes. Notably, as surgeons gain experience, the consistency in surgery duration and low rate of complications underscore the sustained advantages of robotic assistance. Looking ahead, the refinement of these robotic systems and their broader use in thyroid operations are set to push the boundaries of surgical capabilities further, prioritizing the enhancement of patient care and the elevation of surgical expertise (23, 24).

## Data availability statement

The original contributions presented in the study are included in the article/supplementary material. Further inquiries can be directed to the corresponding author.

## Ethics statement

The studies involving humans were approved by Fujian Medical University Union Hospital. The studies were conducted in accordance with the local legislation and institutional requirements. The participants provided their written informed consent to participate in this study.

## Author contributions

BW: Conceptualization, Data curation, Formal Analysis, Funding acquisition, Investigation, Methodology, Supervision, Visualization, Writing – original draft, Writing – review & editing. J-FY: Data curation, Formal Analysis, Methodology, Software, Visualization, Writing – original draft, Writing – review & editing. WA: Conceptualization, Formal Analysis, Writing – review & editing. JW: Data curation, Methodology, Writing – review & editing. X-YG: Data curation, Formal Analysis, Investigation, Writing – review & editing. M-YL: Data curation, Formal Analysis, Writing – review & editing. W-YH: Data curation, Formal Analysis, Methodology, Writing – original draft. C-PZ: Data curation, Formal Analysis, Writing – review & editing. S-YY: Data curation, Formal Analysis, Writing – review & editing. L-YZ: Data curation, Formal Analysis, Writing – review & editing. S-SW: Data curation, Formal Analysis, Writing – review & editing. S-JC: Data curation, Formal Analysis, Writing – review & editing. S-YL: Data curation, Formal Analysis, Writing – review & editing. W-XZ: Conceptualization, Funding acquisition, Investigation, Project administration, Supervision, Writing – review & editing.
